# Adaptive Swarm Balancing Algorithms for rare-event prediction in imbalanced healthcare data

**DOI:** 10.1371/journal.pone.0180830

**Published:** 2017-07-28

**Authors:** Jinyan Li, Lian-sheng Liu, Simon Fong, Raymond K. Wong, Sabah Mohammed, Jinan Fiaidhi, Yunsick Sung, Kelvin K. L. Wong

**Affiliations:** 1 Department of Computer and Information Science, University of Macau, Taipa, Macau SAR; 2 First Affiliated Hospital of Guangzhou University of TCM, Guangzhou, Guangdong, China; 3 School of Computer Science and Engineering, University of New South Wales, Sydney, NSW, Australia; 4 Department of Computer Science, Lakehead University, Thunder Bay, Canada; 5 Computer Engineering Division, Keimyung University, Daegu, South Korea; 6 School of Medicine, University of Western Sydney, Campbelltown, NSW, Australia; Tianjin University, CHINA

## Abstract

Clinical data analysis and forecasting have made substantial contributions to disease control, prevention and detection. However, such data usually suffer from highly imbalanced samples in class distributions. In this paper, we aim to formulate effective methods to rebalance binary imbalanced dataset, where the positive samples take up only the minority. We investigate two different meta-heuristic algorithms, *particle swarm optimization* and *bat algorithm*, and apply them to empower the effects of synthetic minority over-sampling technique (SMOTE) for pre-processing the datasets. One approach is to process the full dataset as a whole. The other is to split up the dataset and adaptively process it one segment at a time. The experimental results reported in this paper reveal that the performance improvements obtained by the former methods are not scalable to larger data scales. The latter methods, which we call *Adaptive Swarm Balancing Algorithms*, lead to significant efficiency and effectiveness improvements on large datasets while the first method is invalid. We also find it more consistent with the practice of the typical large imbalanced medical datasets. We further use the meta-heuristic algorithms to optimize two key parameters of SMOTE. The proposed methods lead to more credible performances of the classifier, and shortening the run time compared to brute-force method.

## Introduction

Big Data in medical fields, such as hospital informatization construction, the progress of treatments, and the extensive use of high-throughput equipment, have caused a geometric growth of attentions. It has been desirable to improve the efficiency, accuracy and quality of medical data processing [1]. The sources of health data include clinical medical treatments, pharmaceutical companies, medical research, medical assistance application, and more. Existing datasets bring in important medical and health information for research topics, such as understanding of the human genetic and disease systems [2] [3], medical and biological imaging [4]; and classification and prediction in medical engineering [5].

Specifically, we investigate disease diagnosis in the context of data mining and classification. Disease diagnosis can be divided into two stages: we first obtain the diagnostic rules from clinical data with known labels, and then apply the rules to diagnosis new patients. However, the high complexity, heterogeneous sources and uncertain reliabilities of medical data pose challenges for classification. For example, it is well known that compared with normal and healthy persons, patients comprise only a small part of the total population. Those more serious diseases, such as cancer and AIDS, have fewer numbers of cases. That constitutes the imbalanced dataset when we try to train classifiers on such data, which causes over-fitting the majority classes and biases our results For instance, in the binary classification of a cancer dataset, the amount of the negative samples (healthy) is dominant, and the obtained model is likely to have little discriminative ability on the positive samples (patient). However in practice, it is an unacceptable mistake to identify cancer patients as healthy people.

In our experiments to solve the imbalanced dataset classification problem, we combine SMOTE and meta-heuristic algorithms to created two methods, which respectively process the data as a whole and partition it into segments. The first method is simple parameter optimization of SMOTE by the meta-heuristic algorithms, namely the Swarm Balancing Algorithms, and after the experiments, we find that it is effective in processing a static and relatively small imbalanced dataset. However the experimental results reflect that the effect of first method is not very good for handling big and highly imbalanced dataset. Therefore, the big data will be divided into several data segments (We used the concept of windows to name these several data segments) suitable for processing by the next methods. To perform SMOTE and classification, the parameters of the data in each window are established on the basis of last window, and the algorithm eventually collects the performances of each window and determines their average values. We call this method Adaptive Swarm Balancing Algorithms in this paper. We observed in our experiments that this latter method is faster and more efficient.

## Related work

In recent years, more and more researchers from different fields have begun to focus on imbalanced dataset research. This research can be considered as having two different levels, the first concerns methods of data modification and optimization, and the second relates to improvement of the algorithms.

### Data level methods

Random under-sampling [6] is a simple sampling technique in which parts of the majority class data are randomly removed to reduce the imbalance ratio, i.e., the ratio between minority and majority is not equal to one but with this method, it is easy to ignore the useful information in the majority class. Contrary to the under-sampling method, random over-sampling, as the other sampling technique [7], increases the number of minority class data to improve the imbalance problem of the dataset. However, its disadvantage is its focus on classification over learning [8]. Based on the over-sampling technique, the synthetic minority oversampling technique (SMOTE) algorithm [9] is a commonly used algorithm that often obtains excellent results in imbalanced dataset classification. The principle is the algorithm is to analyze the feature space of the minority class samples, then synthesize the minority class data and combine it with the original dataset to reduce the imbalance ratio. Assuming that the oversampling rate is *S*, the number of minority classes is equal to *M*, and each minority class can be signified as *x*_*i*_ (*i* = 1, 2, 3 … *M*), which belongs to *S*_*min*_, then every *x*_*i*_ searches out *K* neighbors of the minority class, and the algorithm randomly sets an *x*_*t*_ from the *K* neighbors and finally synthesizes new data:

$$x_{new}=x_{i}+rand\left[0,1\right]\;*\;\left(x_{t}\text{-}x_{i}\right)$$ (1)

Eq (1) synthesizes *S* times new samples, and the function *rand* [0, 1] produces a random number in the scope of 0 to 1. The two key parameters of this algorithm, *S* and *K*, influence the data synthesis and the classification performance. In our experiment, we use meta-heuristic algorithms to find the best and most suitable parameters for the SMOTE algorithm.

### Algorithm level methods

The research emphasis in imbalanced dataset classification is the minority class data. It is more meaningful that the algorithm correctly identifies the minority class rather than the majority class samples. In other words, the cost is higher if the classification algorithm misclassifies the minority class data. The cost-sensitive learning [8] approach assigns different error prices to different classes. If a classifier misclassifies a minority class, it is “punished” in a manner that forces the classifier to increase its recognition rate of minority class samples. Meanwhile, on the basis of the kernel processing method, researchers modified the support vector machine classification in the field of machine learning, which also improved the imbalanced dataset classification problem [10]. The idea of ensemble learning methods is to use an algorithm to obtain a series of child classifiers from the training set and then by integrating these child classifiers, to improve the classification accuracy. SMOTEBoost [11] is an algorithm that combines the SMOTE and Boosting methods; it is a quite effective method among the ensemble learning methods.

In recent year, swarm intelligence algorithms are widely used in different fields to solve the rough original dataset, especially feature selection [12, 13]. We use two different meta-heuristic algorithms, particle swarm optimization (PSO) [14] and the bat algorithm (BA) [15] for comparison of the optimization effect between the two different meta-heuristic algorithms. We choose the neural network algorithm, a representative and popular intelligence classification algorithm, for verification of the classification performance in each iteration.

Pseudo code of PSO:

**For** *each particle*

    *Initialize particle and parameters*

**End**

**While** *maximum iterations or the termination mechanism is not satisfied*.

    **For** *each particle*

        *Calculate and update particle velocity and position as equation*

    *(2) and (3)*

    **End**

    **For** *each particle*

        *Calculation of fitness function*

        **If** *the fitness value is better than the best fitness value (pBest) in*

        *history*

            *current fitness value represent the older pBest to be the new*

              *pBest*

    **End**

  **End**

  *Selected the gBest whose fitness value is the best in the population*.

**End**

PSO [15] is a widely used meta-heuristic algorithm that imitates the feeding process of birds. Above-mentioned pseudo code describe the process of PSO. Assuming there is a population *X* = (*X*_1_, *X*_2_,…, *X*_*n*_) which is grouped by *n* particles in *D* dimension search space, the *i*^th^ particle in this space is expressed as a vector *X*_*i*_ with *D* dimension, *X*_*i*_ = (*x*_*i*1,_ *x*_*i*2_, …, *x*_*iD*_)^*T*^, and the position of the *i*^th^ particle in the search space represents a potential solution. As the objective function, the program can calculate the corresponding fitness of position *X*_*i*_ of each particle, where the speed of the *i*^th^ particle is *V*_*i*_ = (*V*_*i*1_,*V*_*i*2_, …, *V*_*iD*_)^*T*^, the extremum value of each agent is *P*_*i*_ = (*P*_*i*1_, *P*_*i*2_, …, *P*_*iD*_)^*T*^ and the extremum of the population is *P*_*g*_ = (*P*_*g*1_, *P*_*g*2_, …, *P*_*gD*_)^*T*^. In the process of iteration, the extremum value of each agent and the population will update their position and speed [16]. Eqs 2 and 3 show the mathematical process as follows:

$$V_{id}^{k+1}=\omega V_{id}^{k}+c_{1}r_{1}(P_{id}^{k}-X_{id}^{k})+c_{2}r_{2}(P_{gd}^{k}-X_{id}^{k})$$ (2)

$$X_{id}^{k+1}=X_{id}^{k}+V_{id}^{k+1}$$ (3)

In Eq 2, *ω* is inertia weight; *d* = 1, 2, …, *D*; *i* = 1, 2, …, *n*; *k* is the current iteration time; *c*_1_ and *c*_2_ are non-negative constants as the velocity factor, *r*_1_ and *r*_2_ are random values between 0 to 1 and *V*_*id*_ is the particle speed [17].

Pseudo code of BA [17]:

**For** *each bat*

    *Initialize bat*

    *Define pulse frequency at this bat*

    *Initialize pulse rates and the loudness*

**End**

**While** *maximum iterations or the termination mechanism is not satisfied*.

    **For** *each bat*

      *Generate new solutions by frequency*, *and updating velocities and*

      *positions as eqs (4)-(6)*

    **End**

    **For** *each bat*

        **If** *rand bigger than pulse rates*

            *Select a solution among the best solutions and generate a local solution around the select best solution*

        **End**

        *Generate a new solution by flying randomly*

         **If** *rand smaller than loudness and the best value bigger than current fitness value*

            *Accept the new solutions and increase pulse rate and reduce*

                *loudness*

        **End**

    **End**

  *Rank the bats and select the best value in the population*.

**End**

The other algorithm, BA [15], is a new meta-heuristic algorithm that has already shown good results in research. Moreover, we also list the pseudo code of BA of aforementioned part. It learns from the theory of echolocation in bats. The algorithm also assumes the bat populations in a *D* dimension search pace, and the following equations show the process of updating the bats’ position *x*_*i*_ and *v*_*i*_, in the *t*^th^ iteration:

$$f_{i}=f_{min}+(f_{max}-f_{min})\beta$$ (4)

$$v_{i}^{t}=v_{i}^{t-1}+(x_{i}^{t}-x*)f_{i}$$ (5)

$$x_{i}^{t}=x_{i}^{t-1}+v_{i}^{t}$$ (6)

In these three equations, *β* is a random vector between 0 to 1, and x_*_ is the current global best solution among the bats.

In recently years, based on the original version, there are many variant versions of PSO and BA to improve their searching ability in accuracy and efficiency, like SEPSO [18], APSO [19]and FDR-PSO [20], as well as Self-Adaptive Bat algorithm [21], hybrid bat algorithm [22] and chaotic bat algorithm [23], etc. In the future we will try to adopt different version of meta-heuristic algorithms to expand our experiments.

## Experiments and datasets

Differences in the sources and formats of datasets cause complexity. In this paper, the health and medical datasets are divided into two kinds according to the size of the datasets, which are processed by the two methods, Swarm Balancing Algorithms and Adaptive Swarm Balancing Algorithms. Therefore, two experiments are performed as follows. The following experimental results responded that the first method is more suitable for the relatively small dataset. However it would be invalid when the processed dataset is relatively big. As above mentioned that big data is common to seen in health care filed and imbalanced classification problem [24]. Therefore, the latter method was proposed to overcome the big and highly imbalanced dataset.

For the optimizer, Table 1 contains the information of operating environments and the parameters of the two swarm algorithms. Since these parameters are susceptible to performance, thus they are carefully selected from several tests. In PSO, the two learning factors, c1 and c2 which were widely used in equal and smaller than 2. BA has more parameters. Loudness and pulse rate can influence the position of bats to search the neighbor of the objectives. Here we separately set the values of these two parameter to 0.5 and 1. What’s more, the other two factors of BA, Qmin and Qmax, which were commonly used the value of 0 and 1. Furthermore, the populations and the amount of iterations of PSO and BA were the same.

**Table 1:** The environment and parmeters of PSO and BA.

PSO		BA	
Parameter	value	Parameter	value
Population	20	Populations	20
Iterations	1000	Iterations	1000
c_1_	1.5	A(loudness)	0.5
c_2_	1.5	R(pulse rate)	1
		Q_min_	0
		Q_max_	2

All the software programs were coded in MATLAB version 2014a, and the computing environment for all experiments was a PC workstation (CPU: E5-2670 V2 @ 2.50 GHz, RAM: 128 GB).

Both of the following two experiments used 10-fold cross validation method to perform the testing experiment. That means a dataset will be split into 10 non-redundant pieces. Each piece of the ten samples and the rest nine parts respectively are used as the testing dataset and training dataset. The algorithms compute the average value of the ten for verifying the performance. In the second experiment we find that the size of testing dataset (one-tenth of the original dataset) is bigger than the whole dataset in the first experiment.

### Experiment 1: Swarm Balancing Algorithms with a moderately imbalanced dataset

We selected five datasets from the UCI [25] in our experiment. The imbalance ratios between majority class and minority class range from 2.05:1 to 70.3:1.The Surgery dataset in S1 Data, contains data on lung surgery for 5 years. Some datasets related to bioassay data are imbalanced datasets. We selected four of them and respectively stored them in S2 Data to S5 Data, for testing the basic method in the first experiment.

The main problem in the classification of an imbalanced dataset is that the algorithm ignores the minority class data and tends to assign the trained classifier to the majority class with very good accuracy. However, the Kappa statistic is an index that can help people to judge the confidence level of the classification results. It is a very important value when judging problems of imbalanced class classification because even though the accuracy may be high, the Kappa [26, 27] for the classification results can be close to zero or sometimes even a negative value. The Kappa index ranges between -1 to 1. As mentioned in the introduction, in disease diagnosis, classifying a patient as normal is completely unacceptable, and the consequences can be tragic.

As a monitor of the credibility of the classification results, a higher Kappa value indicates that the accuracy is more credible. The Kappa index is commonly divided into three levels to evaluate the credibility of classification accuracy [28, 29]. In the top level, the Kappa value is ≥0.75, which means that the classification accuracy is high in credibility. A Kappa value from ≥0.4 to <0.75 indicates general credibility. Finally, a Kappa value of <0.4 indicates a classification accuracy with either low or no credibility. Our aim in this experiment is to ensure relatively high accuracy by maintaining the largest possible Kappa value. In the experimental process, we used PSO and BA to globally search the two best parameters for SMOTE, *K* and *S*, and the neural network classification algorithm to help the meta-heuristics to estimate and check the two objectives in accordance with the fitness in every iteration of the meta-heuristic algorithms. It means that in the experimental process (also the same in experiment 2), accuracy was not the only objective; we also needed to consider the Kappa index as both are our objectives in optimization.

$$\mathrm{Accuracy}=\frac{\mathrm{TP}+\mathrm{TN}}{P+N}$$ (7)

$$\mathrm{Kappa}=\frac{P_{o}\;\text{-}\;P_{c}}{1\text{-}\;P_{c}}$$ (8)

$$P_{o}=\mathrm{Accuracy}=\frac{TP+TN}{P+N}$$ (9)

$$P_{c}=\frac{\left(\mathrm{TP}+\mathrm{FP})\times (\mathrm{TP}+\mathrm{FN})+(\mathrm{FN}+\mathrm{TN})\times (\mathrm{FP}+\mathrm{TN}\right)}{{\left(P+N\right)}^{2}}$$ (10)

$$\mathrm{Kappa}=\frac{\mathrm{Accuracy-}\;P_{c}}{1\text{-}\;P_{c}}$$ (11)

TP means true positive, TN means true negative, FP means false positive, FN means false negative and P and N respectively stand for positive and negative. From the above equations, we can find that Kappa and Accuracy have inevitable links. Thus both Eqs 7 and 8 are our objective functions.

To find a balance between the two, we set a condition for both. Since we knew the credible range of Kappa, therefore we fixed the Kappa value in the top two intervals to ≥0.4 (this value of Kappa can be changed just as with a threshold or parameter value). The swarms regarded Kappa and Accuracy as fitness function to find the optimal position, gradually[30].

**Fig 1:**
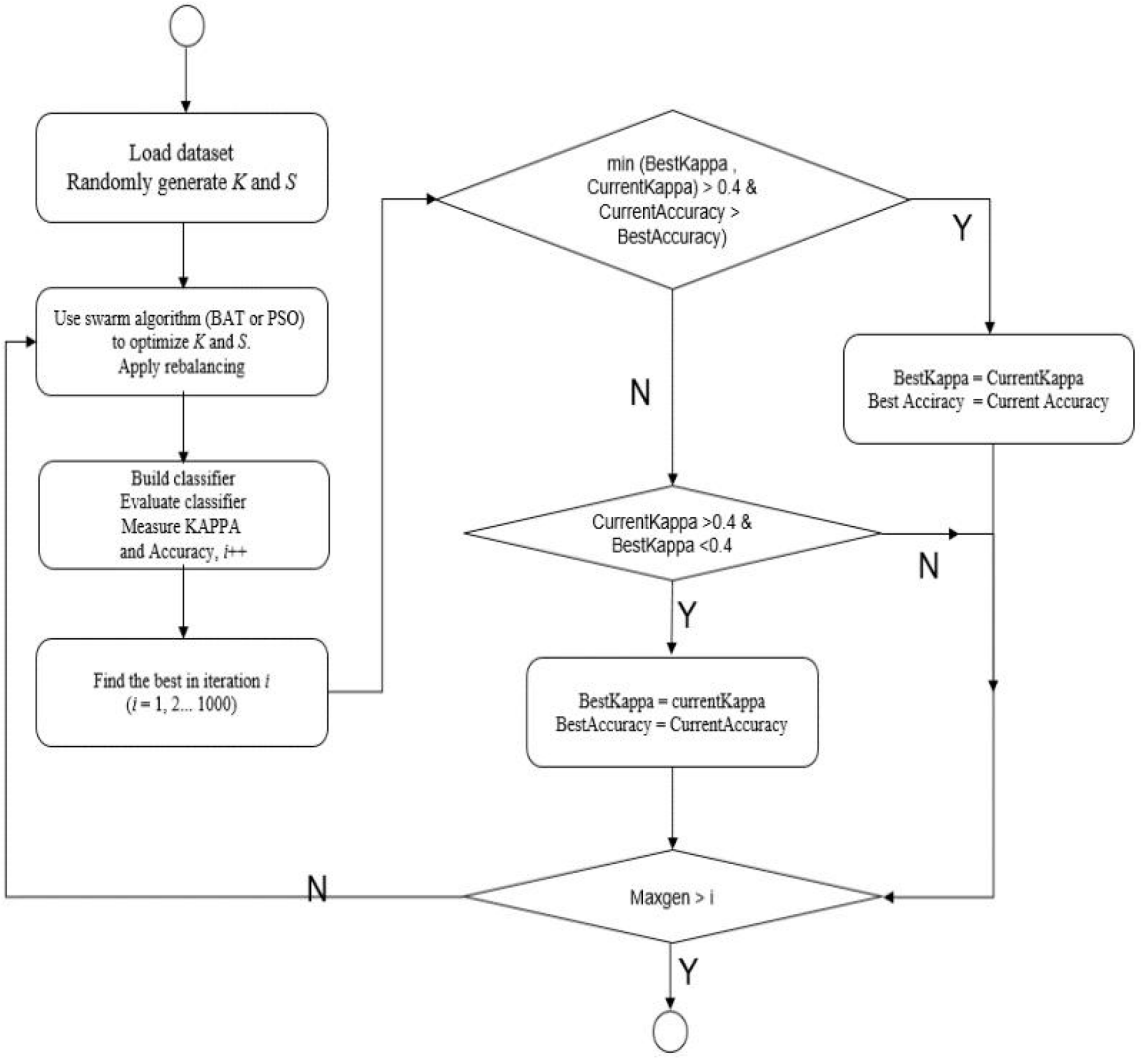
Flow chart of the Swarm Balancing Algorithms.

Fig 1 illustrates the principle and flow chart of the Swarm Balancing Algorithms [30].We initialized two control conditions in the generation of the meta-heuristic algorithms to maintain the authenticity of accuracy, so the first condition was the value of Kappa that must fail in the first and second levels of the Kappa scope (Kappa ≥0.4). Secondly, after satisfying the first condition, the particles or bats needed to find the largest possible accuracy in the search space with control of the two parameters.

Pseudo code of Swarm Balancing Algorithm [30]:

Specify a Meta-heuristic algorithm S (PSO/BA) and a Classifier C (Neural Network)

*Initialize the population of the S algorithm p*_*a*_ *(a = 1*, *2*, *…*, *P) and the other related parameters*

Initialize the floor value of Kappa

Define the scope of K and S

*//K ϵ [K*_*Min*,_ *K*_*max*_*]*, *S ϵ [S*_*Min*,_ *S*_*max*_*]*, *K is the selected Neighbor and S is the increased proportion of minority class //data*

Define the limit value of Kappa T

Load dataset

**While** *(i < Maximum number of iterations)*

    **If** *(i = 1) // in S* then

        *K = Rnd (K*_*i*_ *)*

        *S = Rnd (S*_*i*_ *)*

        *// as initialized parameters of SMOTE to generate a new dataset and using C to get the Current Kappa*

        *//and Current Accuracy*

    **Else**

        *based on the last position or solution to generate a pair of K and S*

          *// through the SMOTE and C to get the Current Kappa and Current Accuracy*

    **End**

    **If** *(min (BestKappa*,*CurrentKappa) > 0*.*4 & CurrentAccuracy > BestAccuracy)* then

            *BestKappa = Current Kappa*

            *BestAccuracy = CurrentAccuracy*

    **Else** *(CurrentKappa >0*.*4 & BestKappa <0*.*4) then*

            *BestKappa = Current Kappa*

            *BestAccuracy = CurrentAccuracy*

    **End**

    *i = i+1*

**End**

In general, the Kappa value increases while accuracy rises. The interval of *S* is from 10% to the value of the number of majority classes divided by minority classes, and the scope of *K* is from 2 to the number of minority classes. To realize the effect of our method, we used normal SMOTE for compression, and in the experiment, we used SMOTE to synthesize minority class samples until the number of minority class data were equal to that of the majority class to obtain a compete balance of the dataset; meanwhile, we used the default value of *K* which is 5. Furthermore, a contrast test was performed using the traditional class balancing algorithm on the same imbalanced datasets, on which the neural network was also used as the classification algorithm for verification. The principle of this algorithm is to change an imbalanced dataset into a completely balanced dataset by splitting the majority class into minority classes.

### Experiment 2: Adaptive Swarm Balancing Algorithms with highly imbalanced dataset

AID 362 dataset (it’s in S5 Data) in the first experiment and the other five highly imbalanced datasets (they are in S6 Data to S10 Data) are also selected from the Bioassay multiple dataset in UCI [25], and they were used in this experiment. However, regardless of the number of features or the imbalance ratio of these datasets, they are much larger than the datasets used in experiment 1. Compared with the datasets in the last experiments, the datasets in this experiments have increased not only in overall size but also in the scales of minority class dataset growth. Therefore, we treated them as big data in our experiments. The largest dataset has 47,831 data instances.

Table 2 lists the characteristics of these highly imbalanced datasets, which are high in volume and dimensions. The approach of Adaptive Swarm Balancing Algorithms is to process the full dataset window by window or to break up the big data into several parts that imitate the data flow to improve the imbalanced dataset classification problem. In our experiment, due to considerations of data size and volume and to guarantee the integrity of the original dataset, we used three windows for each dataset (the concept mentioned in section I) when performing this experiment. Table 2 also shows the length of each window, which indicates how many instances of the dataset are present in each window.

**Table 2:** Characteristics of the highly imbalanced datasets used in experiment 2.

Bioassay No.	Negative	Length of each window in *N*	Positive	Length of each window in *N*	Total instances	Imbalance ratio (Majority/Minority)
362	3375	562(+1), 1124(+1), 1686(+1)	48	8, 16, 24	3423	70.3125
1608	772	128(+1), 256(+1), 384(+2)	55	9, 18, 27(+1)	827	14.03636364
746	47538	7923, 15846, 23769	293	48(+1), 96(+2), 144(+2)	47831	162.2457338
687	26378	4396, 8792(+1), 13188(+1)	76	12(+1), 24(+1), 36(+2)	26454	347.0789474
456	7964	1327, 2654(+1), 3981(+1)	22	3(+1), 6(+1), 9(+2)	7986	362
373	47781	7963(+1), 15926(+1), 23889(+1)	50	8, 16(+1), 24(+1)	47831	955.62

**Fig 2:**
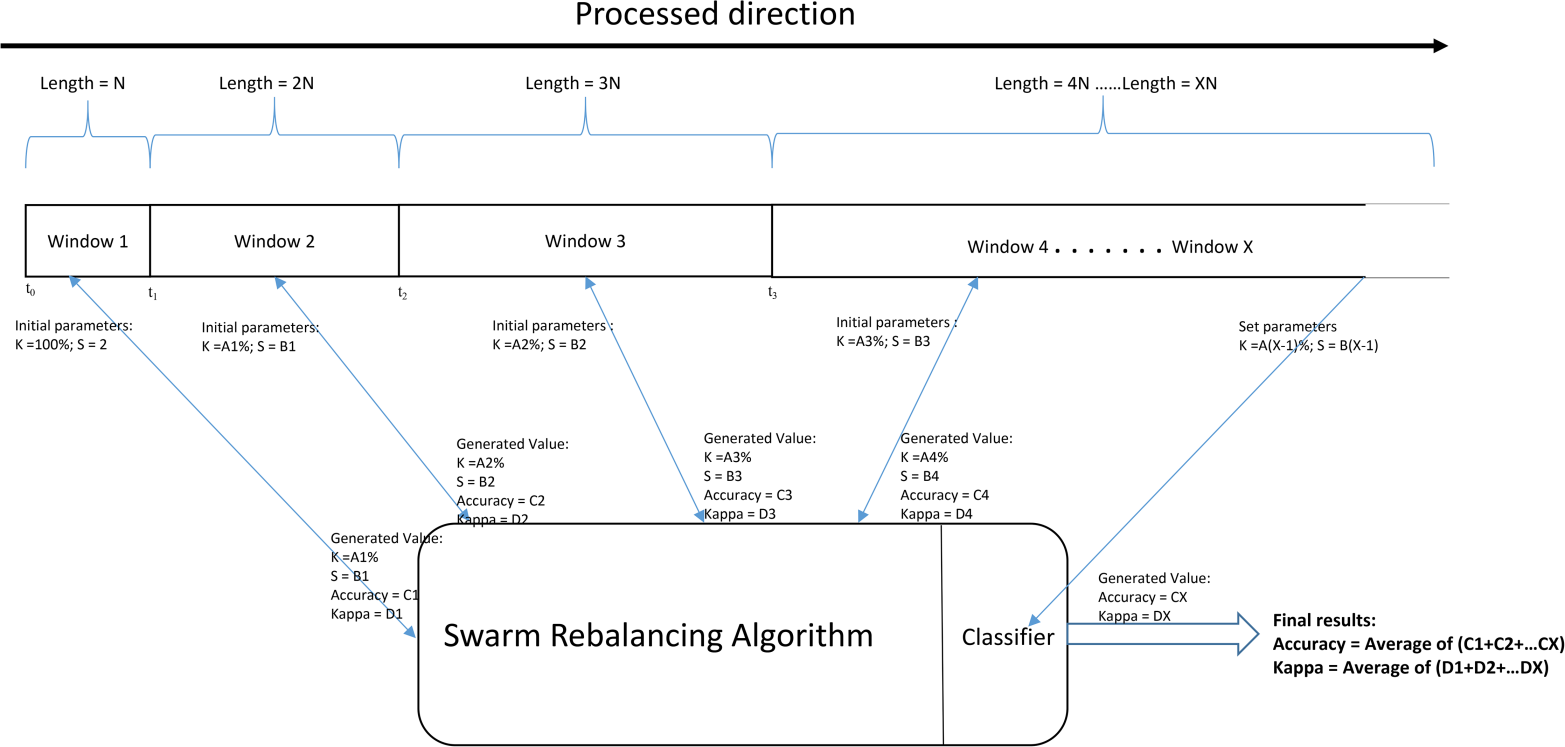
Principle of the Adaptive Swarm Balancing Algorithms.

The principle or working flow of the Swarm Balancing Algorithm is presented in Fig 2, which clearly shows the important role of this algorithm in the experiment. Each of the data windows needs to use this method. From the figure, we can see that the length of Window *X* is *X* times longer than Window 1, which means that as the data flows, the size of the data or the window becomes longer and longer. In Window 1, the initial parameters input into the Swarm Balancing Algorithms are *K* = 100% and *S* = 2, and the algorithms will process the child dataset in Window 1 and generate a suitable *K* = *A*1% and *S* = *B*2 with the current Accuracy and Kappa values.

Then, the *A*1% of *K* and the *B*2 of *S* will be regarded as the initial parameters for Window 2 to repeat the process, which also generates the present values of *K*, *S*, Accuracy and Kappa. This process is repeated until the last window, Window *X*. Here, the algorithm need only use the parameters generated from Window (*X*-1) as its setting parameters to perform the classification. The algorithm ultimately determines the average of each window’s Accuracy and Kappa as the final result. We can find that the processing direction and the data segment direction are opposite. Following is the pseudo code of the Adaptive Swarm Balancing Algorithm which is the novel method which is proposed in this paper:

Pseudo code of Adaptive Swarm Balancing Algorithm:

Specify a Meta-heuristic algorithm S (PSO/BA/…) and a Classifier C (Neural Network/Decision Tree/…)

*Initialize the population of the S algorithm p*_*a*_ *(a = 1*, *2*, *…*, *P) and the other related parameters*

initialize the floor value of Kappa

*Initialize the number of windows as N (x = 1*,*2*,*…N))*

Define the scope of K and N

*//K ϵ [K*_*Min*,_ *K*_*max*_*]*, *S ϵ [S*_*Min*,_ *S*_*max*_*]*, *K is the selected Neighbor and S is the increased proportion of minority class //data*

Define the limit value of Kappa T

Load dataset and divide into x windows

**For** x = 1 : N

    **If** *(x = 1)* then

        *K*_*x*_ *= Rnd (K) // K from 2*

        *S*_*x*_ *= Rnd (S) // S from 100*

        *// as initialized parameters of SMOTE to generate a new dataset and using C to get the Current Kappa*

        *//and Current Accuracy*

    **Else**

        *K*_*x*_ *= K*_*x-1*_ *// K from the final results or last Window x-1*

        *S*_*x*_ *= S*_*x-1*_ *// S from the final results or last Window x-1*

    **End**

    If (x ! = N)

      **While** *(i < Maximum number of iterations)// input the current child dataset*, *current K and S into Swarm Balancing*

          **If** *(i = 1) S* then

            *K*_*xi*_ *= K*_*x*_

            *S*_*xi*_ *= S*_*x*_

            *// as initialized parameters of SMOTE to generate a new dataset and using C to get the Current Kappa*

            *//and Current Accuracy*

          **Else**

           *based on the last position or solution to generate a pair of K and S*

            *// through the SMOTE and C to get the Current Kappa and Current Accuracy*

          **End**

          **If** *(min (BestKappa*,*CurrentKappa) > 0*.*4 & CurrentAccuracy > BestAccuracy)* then

            *BestKappa = Current Kappa*

            *BestAccuracy = CurrentAccuracy*

          **Else** *(CurrentKappa >0*.*4 & BestKappa <0*.*4) then*

            *BestKappa = Current Kappa*

            *BestAccuracy = CurrentAccuracy*

          **End**

          *i = i+1*

          return *K*_*x*_, *S*_*x*,_ *BestAcurracy*_*x*_, *BestKappa*_*x*_

        **End**

    **Else**

        *input K*_*x-1*_ *and S*_*x-1*_ *into SMOTE to generate a new dataset and using C to get the Current Kappa*

        return *BestAcurracy*_*x*_, *BestKappa*_*x*_

    ***E*nd** if

**End** for

***Final Accuracy*** *= average (BestAccuracy*_*1*_, *BestAccuracy*_*2*_, *…*, *BestAccuracy*_*n*_*)*

***Final Kappa**** = average (BestKappa*_*1*_, *BestKppa*_*2*_, *…*, *BestKappa*_*n*_*)*

In experiment 2 we also used SMOTE to synthesize the minority class samples until we obtained a compete balance of the dataset with the default value of *K* = 5 to do the contrast test. As mentioned above that the method of 10-fold cross-validation are used to verify the experimental results.

## Results and discussion

### Results of experiment 1

Our experiment collected performance results in terms of Accuracy, Kappa (Kappa statistic), Precision, Recall, F-measure, ROC area and the imbalance ratio between minority class and majority class. These results are presented in Table 3 to Table 7 with the different classification algorithms or data imbalance processing method, respectively.

**Table 3:** Results of surgery dataset in experiment 1.

Data name:	Surgery Data								
Algorithms	positive	negative	Accuracy	Kappa	Imbalance ratio (Min/Maj)	Precision	Recall	F-Measure	ROC Area
PN	70	400	83.19%	0.00	0.18	0.75	0.83	0.78	0.64
Class balancer-PN	235	235	58.89%	0.18	1.00	0.59	0.59	0.59	0.61
SMOTE(complete balance, K = 5)	399	400	72.09%	0.442	1.00	0.734	0.721	0.717	0.774
PSO-Balancing Algorithm-PN	408	400	82.55%	0.65	1.02	0.83	0.83	0.83	0.85
BA-Balancing Algorithm-PN	213	400	78.14%	0.52	0.53	0.78	0.78	0.78	0.78

**Table 4:** Results of bioassay 439 dataset in experiment 1.

Data name:	AID439								
Algorithms	positive	Negative	Accuracy	Kappa	Imbalance ratio (Min/Maj)	Precision	Recall	F-Measure	ROC Area
PN	11	45	73.21%	0.12	0.24	0.72	0.73	0.73	0.69
Class balancer-PN	28	28	49.29%	-0.01	1.00	0.49	0.49	0.48	0.46
SMOTE(complete balance, K = 5)	44	45	79.78%	0.60	0.98	0.842	0.798	0.791	0.774
PSO-Balancing Algorithm-PN	34	45	82.28%	0.65	0.76	0.86	0.82	0.82	0.87
BA-Balancing Algorithm-PN	40	45	78.82%	0.58	0.89	0.81	0.79	0.79	0.80

**Table 5:** Results of bioassay 721 dataset in experiment 1.

Data name:	AID721								
Algorithms	positive	Negative	Accuracy	Kappa	Imbalance ratio (Min/Maj)	Precision	Recall	F-Measure	ROC Area
PN	17	59	78.95%	0.09	0.29	0.83	0.79	0.71	0.41
Class balancer-PN	38	38	40.88%	-0.18	1.00	0.62	0.68	0.65	0.49
SMOTE(complete balance, K = 5)	58	59	65.81%	0.32	0.98	0.775	0.658	0.619	0.682
PSO-Balancing Algorithm-PN	63	59	70.49%	0.40	1.07	0.40	0.41	0.40	0.39
BA-Balancing Algorithm-PN	63	59	69.67%	0.38	1.07	0.44	0.46	0.41	0.46

**Table 6:** Results of bioassay 1284 dataset in experiment 1.

Data name:	AID1284								
Algorithms	positive	negative	Accuracy	Kappa	Imbalance ratio (Min/Maj)	Precision	Recall	F-Measure	ROC Area
PN	46	244	84.14%	0.00	0.19	0.71	0.84	0.77	0.52
Class balancer-PN	145	145	50.62%	0.01	1.00	0.51	0.51	0.48	0.50
SMOTE(complete balance, K = 5)	243	244	64.07%	0.28	1.00	0.76	0.641	0.594	0.691
PSO-Balancing Algorithm-PN	202	244	70.32%	0.38	0.83	0.49	0.70	0.58	0.64
BA-Balancing Algorithm-PN	254	244	67.07%	0.33	1.04	0.78	0.67	0.63	0.68

**Table 7:** Results of bioassay 362 dataset in experiment 1.

Data name:	AID362								
Algorithms	positive	negative	Accuracy	Kappa	Imbalance ratio(Min/Maj)	Precision	Recall	F-Measure	ROC Area
PN	48	3375	98.60%	0.00	0.01	0.97	0.99	0.98	0.58
Class balancer-PN	1711.5	1711.5	57.92%	0.16	1.00	0.63	0.58	0.53	0.58
SMOTE(complete balance, K = 5)	3374	3375	63.14%	0.2628	1.00	0.786	0.631	0.574	0.641
PSO-Balancing Algorithm-PN	3393	3375	63.18%	0.26	1.01	0.79	0.63	0.57	0.64
BA-Balancing Algorithm-PN	3344	3375	62.91%	0.26	0.99	0.79	0.63	0.57	0.64

In Table 3, which shows the results from the Surgery dataset, the imbalance ratio (min/maj) in th eoriginal dataset is low, and the two key performance measures of Accuracy and Kappa are at the two extremes of low accuracy, both with Kappa values of zero, which means the classifier results are not credible. The performance of the Swarm Balancing Algorithm showed that our method pulls the classification results into a reliable range of scope, although the accuracy suffers slightly. With the imbalance ratio index, we can observe changes in the degree of imbalance of a dataset, which shows whether our methods need to bring the dataset into a completely balanced state. The performance of SMOTE in processing the completely balanced data is also worse than that with the Swarm Balancing Algorithm. The other four datasets are subsets of a large and diversified bioassay dataset. For the Bioassay dataset AID439, Table 4 clearly shows that our method is better than the traditional classbalancer method, which is used as a comparison benchmark. Our approach simultaneously improves the performance of both Accuracy and Kappa. We find that the PSO Balancing approach systhesizes fewer of the minority class data to obtain better performance than the traditional SMOTE approach. The results for Bioassay dataset AID721 are shown in Table 5. It is hard to attain a Kappa value >0.4 with the two meta-heuristic algorithms, and although the PSO and BA are almost equally effective in achieving a compeletly balanced dataset, their performance is still better than that of SMOTE. The results reflect that for this and the previous datasets, most of the time, PSO is slightly better than BA in finding the two parameters with which to acheive higher performance, but it also needs to synthesize more minority samples. The results in Table 6 also prove the better ability of the Swarm Balancing Algorithms to process the classification of an imbalanced dataset. The results of the last Bioassay dataset, AID362, show the highest imbalance of the five datasets. Thus, it is doubtful that high accuracy with a low Kappa value can be attained with the original classification, and the results are still not good after the orginal dataset is processed by the class balancer method. The performance of the neural network algorithm also remains poor as it does not result in a Kappa value high enough to reach the credible stage of ≥0.4. Meanwhile, the traditional SMOTE shows almost the same performance as that of the Swarm Balancing Algorithms.

**Fig 3:**
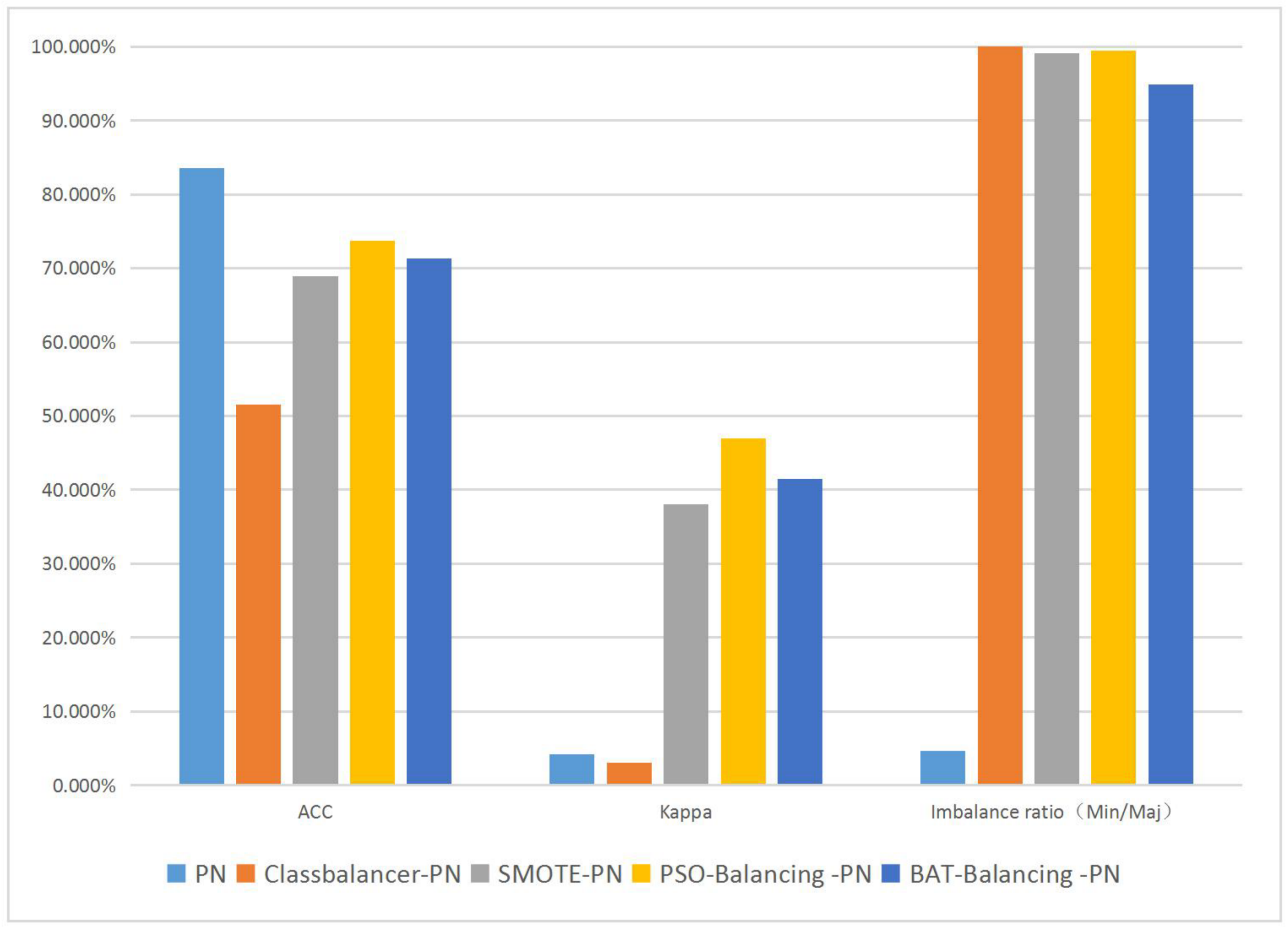
Average performance results of each method in experiment 1.

The bar diagrams in Fig 3 clearly illustrate the contrast in the average values of the different methods that are used in experiment 1 and presented in Tables 3 to 6. We can find that although the class balancing method can bring the dataset into full balance, the performance still very poor.

The performance of the neural network algorithm also remains poor as it does not result in a Kappa value high enough to reach the credible stage of ≥0.4.For the other complete datasets artificially generated by traditional SMOTE, the performance is much better than that with the original and class balancing methods, but there is still a gap with the two Swarm Balancing Algorithms in terms of the two important indexes. Although both the PSO and BA can search the suitable parameters to make the Kappa value fall within the range of credibility, the PSO-Balancing algorithm is better than the BA-Balancing algorithm for both Accuracy and Kappa. From the perspective of the quantity of synthesis necessary, the BA-Balancing algorithm produces less synthesis of minority class samples than the PSO-Balancing algorithm does. As the results in this experiment, the Swarm Balancing Algorithms satisfied the expected goal, which is to attain the highest possible accuracy with a Kappa value falling within a credible and reasonable area. However, as the results from the AID362 dataset show, when this method meets a large and highly imbalanced dataset, the performance is not as good as that in the small datasets, in which a Kappa value of ≥0.4 cannot be reached. Hence, to ensure that our basic concepts are effective in highly imbalanced and larger datasets, we realize that the algorithm needs to be improved.

### Results of experiment 2

All of the results from experiment 2 are listed in Tables 8–14. They include the performance results of Accuracy, Kappa (Kappa statistic), TPR (true positive rate), FPR (false positive rate), Precision, Recall, F-measure, ROC area, the imbalance ratio between minority class and majority class and Searching time.

**Table 8:** Average values of Accuracy, Kappa and imbalance ratio (min/maj) for the two methods in experiment 2.

Neural Network	Acc.	Kap.	Imb. Ratio	Neural Network	Acc.	Kap.	Imb. Ratio
Original data	98.45%	0.000	0.004	Original data	98.45%	0.000	0.004
SMOTE (complete balance, *K* = 5)	63.07%	0.26140	1.00	SMOTE (complete balance, *K* = 5)	63.07%	0.26140	1.00
PSO-Balancing Algorithm	61.73%	0.239	0.790	BA-Balancing Algorithm	62.56%	0.252	0.877
APBA-Window 1	98.34%	-0.002	0.004	ABBA-Window 1	98.34%	-0.002	0.004
APBA-Processed State 1	78.41%	0.565	0.861	ABBA-Processed State 1	79.20%	0.590	0.816
APBA-Window 2	98.41%	-0.001	0.004	ABBA-Window 2	98.41%	-0.001	0.004
APBA -Initial State = APBA -PS1	72.87%	0.408	0.862	ABBA-Initial State = ABSB-PS1	71.41%	0.423	0.822
APBA-Processed State 2	74.13%	0.481	0.974	ABBA-Processed State 2	75.58%	0.505	0.979
APBA-Window 3	98.43%	0.000	0.004	ABBA-Window 3	98.43%	0.000	0.004
APBA-Processed State 1	68.69%	0.292	0.827	ABBA-Processed State 1	67.22%	0.315	0.783
APBA-Processed State 2	69.70%	0.386	0.972	ABBA-Processed State 2	70.71%	0.397	0.977
APBA-Finally Average results	74.08%	0.477	0.936	ABBA-Finally Average results	75.17%	0.497	0.924

**Table 9:** Results of Bioassay 456 dataset in experiment 2.

Data Name:	AID 456												
Neural Network	Percentage	Nearest Neighbors	Accuracy	Kappa	Psize	Nsize	Searching Time(s)	TPR	FPR	Precision	Recall	F-Measure	ROC Area
*Original data*			*99*.*72%*	*0*.*000*	*22*.*000*	*7964*.*000*		*0*.*997*	*0*.*997*	*0*.*994*	*0*.*997*	*0*.*996*	*0*.*512*
*SMOTE(complete balance*, *K = 5)*	36ss1	5	69.64%	0.393	7964.000	7964.000		0.696	0.304	0.811	0.696	0.666	0.710
*PSO-Balancing Algorithm*	*26247*	*21*	*64*.*88%*	*0*.*353*	*5796*.*000*	*7964*.*000*	*406*.*268*	*0*.*649*	*0*.*256*	*0*.*808*	*0*.*649*	*0*.*624*	*0*.*706*
APBA-Window 1			99.70%	0.000	4.000	1327.000		0.997	0.997	0.994	0.997	0.995	0.415
APBA-Processed State 1	27046	3	97.64%	0.953	1085.000	1327.000	121.900	0.976	0.019	0.978	0.976	0.976	0.986
APBA-Window 2			99.74%	0.000	7.000	2655.000		0.997	0.997	0.995	0.997	0.996	0.634
APBA-Initial State = APSB-PS1	27046	3	92.27%	0.845	1900.000	2655.000		0.923	0.055	0.935	0.923	0.923	0.960
APBA-Processed State 2	34165	4	93.03%	0.861	2398.000	2655.000	119.471	0.930	0.063	0.939	0.930	0.930	0.947
APBA-Window 3			99.72%	0.000	11.000	3982.000		0.997	0.997	0.994	0.997	0.996	0.463
APBA-Processed State 1	27046	3	77.90%	0.576	2986.000	3982.000		0.779	0.166	0.854	0.779	0.775	0.815
APBA-Processed State 2	34165	4	79.96%	0.603	3769.000	3982.000		0.800	0.190	0.858	0.800	0.792	0.831
APBA-*Finally Average results*			*90*.*21%*	*0*.*806*			*241*.*370*	*0*.*902*	*0*.*091*	*0*.*925*	*0*.*902*	*0*.*899*	*0*.*921*
*BA-Balancing Algorithm*	*28060*	*17*	*66*.*02%*	*0*.*362*	*6195*.*000*	*7964*.*000*	*504*.*073*	*0*.*660*	*0*.*268*	*0*.*791*	*0*.*660*	*0*.*637*	*0*.*712*
ABBA-Window 1			99.70%	0.000	4.000	1327.000		0.997	0.997	0.994	0.997	0.995	0.415
ABBA-Processed State 1	29511	10	97.65%	0.953	1184.000	1327.000	274.540	0.977	0.021	0.978	0.977	0.977	0.986
ABBA-Window 2			99.74%	0.000	7.000	2655.000		0.997	0.997	0.995	0.997	0.996	0.634
ABBA-Initial State = ABSB-PS1	29511	10	92.66%	0.854	2072.000	2655.000		0.927	0.057	0.937	0.927	0.927	0.946
ABBA-Processed State 2	33294	10	93.03%	0.861	2337.000	2655.000	123.385	0.930	0.061	0.939	0.930	0.930	0.943
ABBA-Window 3			99.72%	0.000	11.000	3982.000		0.997	0.997	0.994	0.997	0.996	0.463
ABBA-Processed State 1	29511	10	78.35%	0.581	3257.000	3982.000		0.784	0.177	0.854	0.784	0.778	0.836
ABBA-Processed State 2	33294	10	79.53%	0.597	3673.000	3982.000		0.795	0.189	0.857	0.795	0.788	0.837
ABBA-*Finally Average results*			*90*.*07%*	*0*.*804*			*397*.*925*	*0*.*901*	*0*.*090*	*0*.*925*	*0*.*901*	*0*.*898*	*0*.*922*

the grey part means there is no searching time in this step.

APBA means Adaptive PSO Balancing Algorithm; ABBA means Adaptive BA Balancing Algorithm.

**Table 10:** Results of Bioassay 362 dataset in experiment 2.

Data Name:	AID 362												
Neural Network	Percentage	Nearest Neighbors	Accuracy	Kappa	Psize	Nsize	Searching Time(s)	TPR	FPR	Precision	Recall	F-Measure	ROC Area
Original data			98.60%	0.000	48.000	3375.000		0.986	0.986	0.972	0.986	0.979	0.580
*SMOTE(complete balance*, *K = 5)*	69.3125	5	63.14%	0.263	3374.000	3375.000		0.631	0.369	0.786	0.631	0.574	0.641
*PSO-Balancing Algorithm*	6969	36	63.18%	0.262	3393.000	3375.000	136.673	0.632	0.370	0.787	0.632	0.574	0.643
APBA-Window 1			98.60%	0.000	8.000	563.000		0.986	0.986	0.972	0.986	0.979	0.398
APBA-Processed State 1	4710	2	85.53%	0.716	384.000	563.000	64.781	0.855	0.099	0.893	0.855	0.856	0.894
APBA-Window 2			98.60%	0.000	16.000	1125.000		0.986	0.986	0.972	0.986	0.979	0.555
APBA-Initial State = APSB-PS1			75.40%	0.528	769.000	1125.000		0.754	0.184	0.819	0.754	0.753	0.862
APBA-Processed State 2	9700	610	82.92%	0.627	1568.000	1125.000	26.575	0.829	0.238	0.867	0.829	0.818	0.853
APBA-Window 3			98.60%	0.000	24.000	1687.000		0.986	0.986	0.972	0.986	0.979	0.484
APBA-Processed State 1	4710	2	61.99%	0.237	1154.000	1687.000		0.620	0.374	0.636	0.620	0.623	0.694
APBA-Processed State 2	9700	610	73.30%	0.396	2354.000	1687.000		0.733	0.373	0.817	0.733	0.695	0.727
APBA-*Finally Average results*			80.58%	0.580			91.356	0.806	0.237	0.859	0.806	0.790	0.825
*BA-Balancing Algorithm*	*6867*	*28*	62.91%	0.261	3344.000	3375.000	149.354	0.629	0.367	0.787	0.629	0.571	0.638
ABBA-Window 1			98.60%	0.000	8.000	563.000		0.986	0.986	0.972	0.986	0.979	0.398
ABBA-Processed State 1	4710	2	85.53%	0.716	384.000	563.000	75.465	0.855	0.099	0.893	0.855	0.856	0.894
ABBA-Window 2			98.60%	0.000	16.000	1125.000		0.986	0.986	0.972	0.986	0.979	0.555
ABBA-Initial State = ABSB-PS1			75.40%	0.528	769.000	1125.000		0.754	0.184	0.819	0.754	0.753	0.862
ABBA-Processed State 2	9893	10	85.57%	0.686	1599.000	1125.000	48.299	0.856	0.205	0.884	0.856	0.848	0.876
ABBA-Window 3			98.60%	0.000	24.000	1687.000		0.986	0.986	0.972	0.986	0.979	0.484
ABBA-Processed State 1	4710	2	61.99%	0.237	1154.000	1687.000		0.620	0.374	0.636	0.620	0.623	0.694
ABBA-Processed State 2	9893	10	72.91%	0.381	2400.000	1687.000		0.729	0.385	0.815	0.729	0.688	0.696
ABBA-*Finally Average results*			81.34%	0.594			123.765	0.813	0.230	0.864	0.813	0.797	0.822

the grey part means there is no searching time in this step.

APBA means Adaptive PSO Balancing Algorithm; ABBA means Adaptive BA Balancing Algorithm.

**Table 11:** Results of Bioassay 1608 dataset in experiment 2.

Data Name:	AID 1608												
Neural Network	Percentage	Nearest Neighbors	Accuracy	Kappa	Psize	Nsize	Searching Time (s)	TPR	FPR	Precision	Recall	F-Measure	ROC Area
*Original data*			93.35%	0.000	55.000	772.000		0.933	0.933	0.871	0.933	0.901	0.410
*SMOTE(complete balance*, *K = 5)*	1304%	5	59.33%	0.187	55.000	772.000		0.593	0.407	0.748	0.593	0.518	0.601
*PSO-Balancing Algorithm*	1162	50	56.75%	0.165	694.000	772.000	157.216	0.568	0.397	0.659	0.568	0.515	0.616
APBA-Window 1			92.75%	-0.013	9.000	129.000		0.928	0.935	0.873	0.928	0.900	0.462
APBA-Processed State 1	386	3	75.00%	0.419	43.000	129.000	64.004	0.750	0.269	0.793	0.750	0.763	0.866
APBA-Window 2			93.09%	-0.007	18.000	257.000		0.931	0.935	0.873	0.931	0.901	0.372
APBA-Initial State = APSB-PS1			75.29%	0.165	87.000	257.000		0.753	0.623	0.713	0.753	0.705	0.753
APBA-Processed State 2	1062	19	71.03%	0.448	209.000	257.000	29.576	0.710	0.236	0.824	0.710	0.694	0.754
APBA-Window 3			93.24%	0.000	28.000	386.000		0.932	0.932	0.869	0.932	0.900	0.562
APBA-Processed State 1	386	3	73.95%	0.000	136.000	386.000		0.739	0.739	0.547	0.739	0.629	0.651
APBA-Processed State 2	1062	19	65.22%	0.338	327.000	386.000		0.652	0.295	0.798	0.652	0.618	0.678
APBA-*Finally Average results*			70.42%	0.401			93.580	0.704	0.267	0.805	0.704	0.692	0.766
*BA-Balancing Algorithm*	1398	29	61.00%	0.200	823.000	772.000	172.092	0.610	0.415	0.763	0.610	0.535	0.613
ABBA-Window 1			92.75%	-0.013	9.000	129.000		0.928	0.935	0.873	0.928	0.900	0.462
ABBA-Processed State 1	685	2	81.41%	0.636	70.000	129.000	76.199	0.814	0.101	0.878	0.814	0.818	0.866
ABBA-Window 2			93.09%	-0.007	18.000	257.000		0.931	0.935	0.873	0.931	0.901	0.372
ABBA-Initial State = ABSB-PS1			71.36%	0.305	141.000	257.000		0.714	0.439	0.705	0.714	0.689	0.770
ABBA-Processed State 2	1595	30	75.98%	0.496	305.000	257.000	47.766	0.760	0.284	0.830	0.760	0.739	0.772
ABBA-Window 3			93.24%	0.000	28.000	386.000		0.932	0.932	0.869	0.932	0.900	0.562
ABBA-Processed State 1	685	2	63.31%	0.072	219.000	386.000		0.633	0.572	0.591	0.633	0.571	0.661
ABBA-Processed State 2	1595	30	70.96%	0.375	475.000	386.000		0.651	0.421	0.773	0.651	0.587	0.620
ABBA-*Finally Average results*			*76*.*12%*	*0*.*502*			123.965	*0*.*742*	*0*.*269*	*0*.*827*	*0*.*742*	*0*.*715*	*0*.*753*

the grey part means there is no searching time in this step.

APBA means Adaptive PSO Balancing Algorithm; ABBA means Adaptive BA Balancing Algorithm.

**Table 12:** Results of Bioassay 373 dataset in experiment 2.

Data Name:	AID 373												
Neural Network	Percentage	Nearest Neighbors	Accuracy	Kappa	Psize	Nsize	Searching Time (s)	TPR	FPR	Precision	Recall	F-Measure	ROC Area
*Original data*			99.90%	0.000	50.000	47831.000		0.999	0.999	0.998	0.999	0.998	0.658
*SMOTE(complete balance*, *K = 5)*	95462	5	68.54%	0.371	47831.000	47831.000		0.685	0.315	0.807	0.685	0.651	0.719
*PSO-Balancing Algorithm*	61214	43	65.27%	0.251	30657.000	47831.000	538.195	0.653	0.409	0.645	0.653	0.647	0.720
APBA-Window 1			99.90%	0.000	8.000	7964.000		0.999	0.999	0.998	0.999	0.998	0.592
APBA-Processed State 1	81124	7	74.48%	0.510	6497.000	7964.000	106.964	0.745	0.208	0.837	0.745	0.735	0.831
APBA-Window 2			99.89%	0.000	17.000	15927.000		0.999	0.999	0.998	0.999	0.998	0.455
APBA-Initial State = APSB-PS1			67.12%	0.381	13808.000	15927.000		0.671	0.258	0.784	0.671	0.655	0.462
APBA-Processed State 2	91409	14	69.89%	0.402	15556.000	15927.000	129.514	0.699	0.295	0.776	0.699	0.678	0.732
APBA-Window 3			99.90%	0.000	25.000	23890.000		0.999	0.999	0.998	0.999	0.998	0.639
APBA-Processed State 1	81124	7	77.98%	0.572	17793.000	23890.000		0.780	0.187	0.851	0.780	0.773	0.850
APBA-Processed State 2	91409	14	79.19%	0.587	22877.000	23890.000		0.792	0.199	0.854	0.792	0.784	0.836
APBA-*Finally Average results*			74.52%	0.500			236.478	0.745	0.234	0.822	0.745	0.732	0.800
*BA-Balancing Algorithm*	71076	44	64.29%	0.256	35588.000	47831.000	541.370	0.643	0.392	0.638	0.643	0.638	0.718
ABBA-Window 1			99.90%	0.000	8.000	7964.000		0.999	0.999	0.998	0.999	0.998	0.592
ABBA-Processed State 1	81124	7	74.48%	0.510	6497.000	7964.000	106.964	0.745	0.208	0.837	0.745	0.735	0.831
ABBA-Window 2			99.89%	0.000	17.000	15927.000		0.999	0.999	0.998	0.999	0.998	0.455
ABBA-Initial State = ABSB-PS1			67.12%	0.381	13808.000	15927.000		0.671	0.258	0.784	0.671	0.655	0.462
ABBA-Processed State 2	89403	10	70.12%	0.401	15215.000	15927.000	167.194	0.701	0.301	0.702	0.701	0.700	0.772
ABBA-Window 3			99.90%	0.000	25.000	23890.000		0.999	0.999	0.998	0.999	0.998	0.639
ABBA-Processed State 1	81124	7	77.98%	0.572	17793.000	23890.000		0.780	0.187	0.851	0.780	0.773	0.850
ABBA-Processed State 2	89403	10	79.05%	0.586	22375.000	23890.000		0.791	0.196	0.854	0.791	0.783	0.844
ABBA-*Finally Average results*			74.55%	0.499			274.159	0.746	0.235	0.798	0.746	0.739	0.816

the grey part means there is no searching time in this step.

APBA means Adaptive PSO Balancing Algorithm; ABBA means Adaptive BA Balancing Algorithm.

**Table 13:** Results of Bioassay 687 dataset in experiment 2.

Data Name:	AID 687												
Neural Network	Percentage	Nearest Neighbors	Accuracy	Kappa	Psize	Nsize	Searching Time (s)	TPR	FPR	Precision	Recall	F-Measure	ROC Area
*Original data*			99.71%	0.000	76.000	26378.000		0.997	0.997	0.994	0.997	0.996	0.577
*SMOTE(complete balance*, *K = 5)*	34608	5	60.37%	0.207	26378.000	26378.000		0.604	0.396	0.779	0.604	0.530	0.641
*PSO-Balancing Algorithm*	32368	75	63.30%	0.268	24675.000	4396.000	6813.241	0.633	0.364	0.636	0.633	0.633	0.673
APBA-Window 1			99.71%	0.000	13.000	4396.000		0.997	0.997	0.994	0.997	0.996	0.533
APBA-Processed State 1	27568	11	73.02%	0.483	3596.000	4396.000	146.408	0.730	0.221	0.831	0.730	0.718	0.799
APBA-Window 2			99.72%	0.000	25.000	8793.000		0.997	0.997	0.994	0.997	0.996	0.535
APBA-Initial State = APSB-PS1			66.91%	0.312	6719.000	8793.000		0.669	0.365	0.668	0.669	0.661	0.743
APBA-Processed State 2	31180	12	66.69%	0.324	7820.000	8793.000	926.972	0.667	0.347	0.670	0.667	0.661	0.747
APBA-Window 3			99.71%	0.000	38.000	13189.000		0.997	0.997	0.994	0.997	0.996	0.513
APBA-Processed State 1	27568	11	61.44%	0.186	10513.000	13189.000		0.614	0.436	0.613	0.614	0.593	0.659
APBA-Processed State 2	31180	12	60.14%	0.185	11886.000	13189.000		0.601	0.420	0.609	0.601	0.583	0.662
APBA-*Finally Average results*			66.62%	0.331			1073.380	0.666	0.329	0.703	0.666	0.654	0.736
*BA-Balancing Algorithm*	32368	75	63.30%	0.268	24675.000	26378.000	5139.300	0.633	0.364	0.636	0.633	0.633	0.673
ABBA-Window 1			99.71%	0.000	13.000	4396.000		0.997	0.997	0.994	0.997	0.996	0.533
ABBA-Processed State 1	28295	7	73.35%	0.487	3691.350	4396.000	262.371	0.734	0.224	0.832	0.734	0.720	0.790
ABBA-Window 2			99.72%	0.000	25.000	8793.000		0.997	0.997	0.994	0.997	0.996	0.535
ABBA-Initial State = ABSB-PS1			64.42%	0.332	7098.000	8793.000		0.644	0.287	0.802	0.644	0.611	0.702
ABBA-Processed State 2	34008	8	67.85%	0.363	8526.000	8793.000	1000.736	0.678	0.312	0.806	0.678	0.644	0.732
ABBA-Window 3			99.71%	0.000	38.000	13189.000		0.997	0.997	0.994	0.997	0.996	0.513
ABBA-Processed State 1	28295	7	60.82%	0.213	10790.000	13189.000		0.608	0.393	0.611	0.608	0.609	0.657
ABBA-Processed State 2	34008	8	60.51%	0.216	12961.000	13189.000		0.605	0.388	0.780	0.605	0.534	0.669
ABBA-*Finally Average results*			67.24%	0.355			1263.107	0.672	0.308	0.806	0.672	0.633	0.730

the grey part means there is no searching time in this step.

APBA means Adaptive PSO Balancing Algorithm; ABBA means Adaptive BA Balancing Algorithm.

**Table 14:** Results of Bioassay 746 dataset in experiment 2.

Data Name:	AID 746												
Neural Network	Percentage	Nearest Neighbors	Accuracy	Kappa	Psize	Nsize	Searching Time (s)	TPR	FPR	Precision	Recall	F-Measure	ROC Area
*Original data*			99.39%	0.000	293.000	47831.000		0.994	0.994	0.988	0.994	0.991	0.543
*SMOTE(complete balance*, *K = 5)*	16124	5	57.40%	0.148	47831.000	47831.000		0.763	0.574	0.481	0.577	57.40%	0.148
*PSO-Balancing Algorithm*	13743	129	56.98%	0.132	40559.000	47831.000	15176.904	0.570	0.438	0.569	0.570	0.569	0.607
APBA-Window 1			99.37%	0.000	49.000	7923.000		0.994	0.994	0.987	0.994	0.991	0.585
APBA-Processed State 1	15393	31	64.79%	0.306	7591.000	7923.000	873.725	0.648	0.337	0.795	0.648	0.602	0.706
APBA-Window 2			99.39%	0.000	98.000	15846.000		0.994	0.994	0.988	0.994	0.991	0.589
APBA-Initial State = APSB-PS1			60.25%	0.218	15183.000	15846.000		0.602	0.381	0.780	0.602	0.533	0.630
APBA-Processed State 2	16120	41	61.24%	0.224	15895.000	15846.000	2813.100	0.612	0.389	0.781	0.612	0.544	0.622
APBA-Window 3			99.39%	0.000	147.000	23768.000		0.994	0.994	0.988	0.994	0.991	0.560
APBA-Processed State 1	15393	31	58.89%	0.178	22774.000	23768.000		0.589	0.411	0.589	0.589	0.589	0.632
APBA-Processed State 2	16120	41	60.38%	0.208	23843.000	23768.000		0.604	0.396	0.604	0.604	0.604	0.638
APBA-*Finally Average results*			62.14%	0.246			3686.825	0.621	0.374	0.727	0.621	0.583	0.655
*BA-Balancing Algorithm*	15863	193	57.84%	0.163	46771.000	47831.000	25927.423	0.578	0.415	0.754	0.578	0.493	0.603
ABBA-Window 1			99.37%	0.000	50.000	7923.000		0.994	0.994	0.987	0.994	0.991	0.585
ABBA-Processed State 1	12927	45	62.77%	0.238	6383.000	7923.000	2393.697	0.628	0.393	0.625	0.628	0.624	0.691
ABBA-Window 2			99.39%	0.000	98.000	15846.000		0.994	0.994	0.988	0.994	0.991	0.589
ABBA-Initial State = ABSB-PS1			57.48%	0.138	12766.000	15846.000		0.575	0.437	0.574	0.575	0.574	0.622
ABBA-Processed State 2	15895	55	60.94%	0.222	15675.000	15846.000	5962.276	0.609	0.386	0.781	0.609	0.541	0.617
ABBA-Window 3			99.39%	0.000	147.000	23768.000		0.994	0.994	0.988	0.994	0.991	0.560
ABBA-Processed State 1	12927	45	60.86%	0.212	19149.000	23768.000		0.609	0.396	0.611	0.609	0.609	0.644
ABBA-Processed State 2	15895	55	61.29%	0.226	23512.000	23768.000		0.613	0.387	0.613	0.613	0.613	0.643
ABBA-*Finally Average results*			61.67%	0.229			8355.974	0.617	0.389	0.673	0.617	0.593	0.650

the grey part means there is no searching time in this step.

APBA means Adaptive PSO Balancing Algorithm; ABBA means Adaptive BA Balancing Algorithm.

Similar to the results with the AID362 dataset in experiment 1, the Swarm Balancing Algorithms show poor effect in all five of the big and highly imbalanced datasets, and although the Kappa values are not equal to 0, they still lie within the areas of low or no credibility. However, both Accuracy and Kappa show great improvement when the Adaptive Swarm Balancing Algorithms are used. The results of the changes in the data with the Adaptive Swarm Balancing Algorithms shown in Tables 9 to 14 range from the best to the worst.

Table 8 shows the average performance of Accuracy. Kappa and imbalance ratio (min/maj) from Tables 9 to 14. The data highlighted in bold format are the classification results of the original dataset, Swarm Balancing Algorithms and Adaptive Swarm Balancing Algorithms. The other parts respectively reflect the results of Window 1, Window 2 and Window 3. Because the length from Window 1 to Window 3 becomes longer and longer, the results with the Adaptive Swarm Balancing Algorithms become worse and worse; however, the final results are, on the whole, much better than those with the Swarm Balancing Algorithms. When the algorithms process a big dataset, the traditional SMOTE is slightly better than the Swarm Balancing Algorithms, but the Adaptive BA-Balancing algorithm is better than the traditional SMOTE and the Adaptive PSO-Balancing algorithm for all three performance parameters because it uses less synthetic minority class data and achieves higher accuracy with a higher Kappa value than the latter two. At the same time, it is easy to see that the problem with the AID362 dataset in experiment 1 has been solved in experiment 2, which shows that the Adaptive Swarm Balancing Algorithms are highly effective in processing a large imbalanced dataset. It must be mentioned that the performance with the 746 dataset, which contains the most data of all, is not good due to its large data size. However, we believe that if we choose to use more windows to process this dataset, the results can be improved.

**Fig 4:**
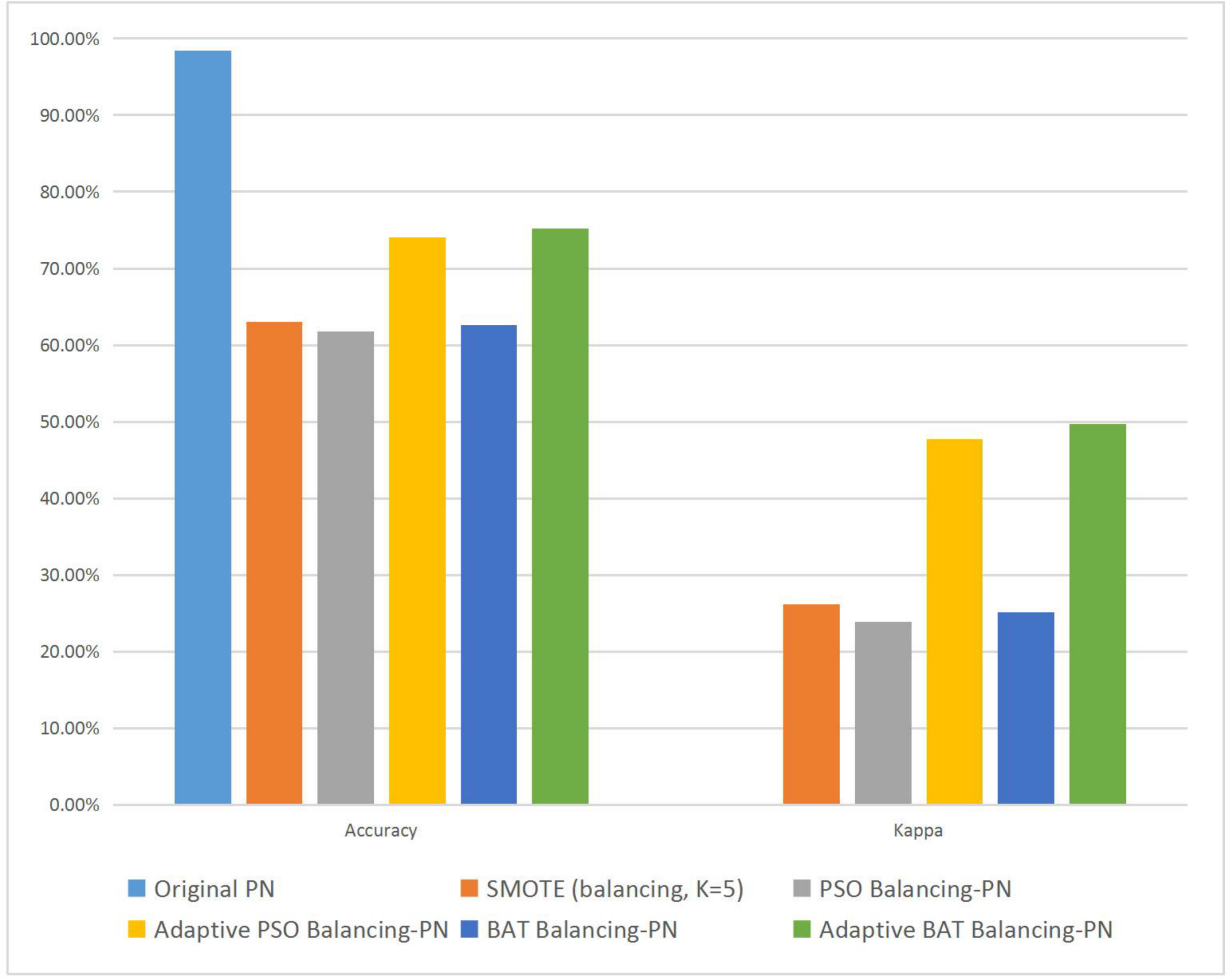
Average Accuracy and Kappa of different methods in experiment 2.

Fig 4 separates the two key performance parameters of Accuracy and Kappa from Table 8 and depicts them graphically in a bar diagram. It is clear that both approaches increase the Kappa value, but that with the Adaptive Swarm Balancing Algorithms is two times higher than that with the Swarm Balancing Algorithms. However, the Kappa value with the latter method still indicates non-credibility, whereas that with the former method indicates credibility and, thereby, higher accuracy. Furthermore, in terms of average values, performance parameters with the traditional SMOTE are much worse than those with the two new Swarm Balancing Algorithms, indicating that optimization of the parameters is more important than rebalancing of the dataset and that a completely balanced dataset does not necessarily mean that a better result can be achieved.

**Fig 5:**
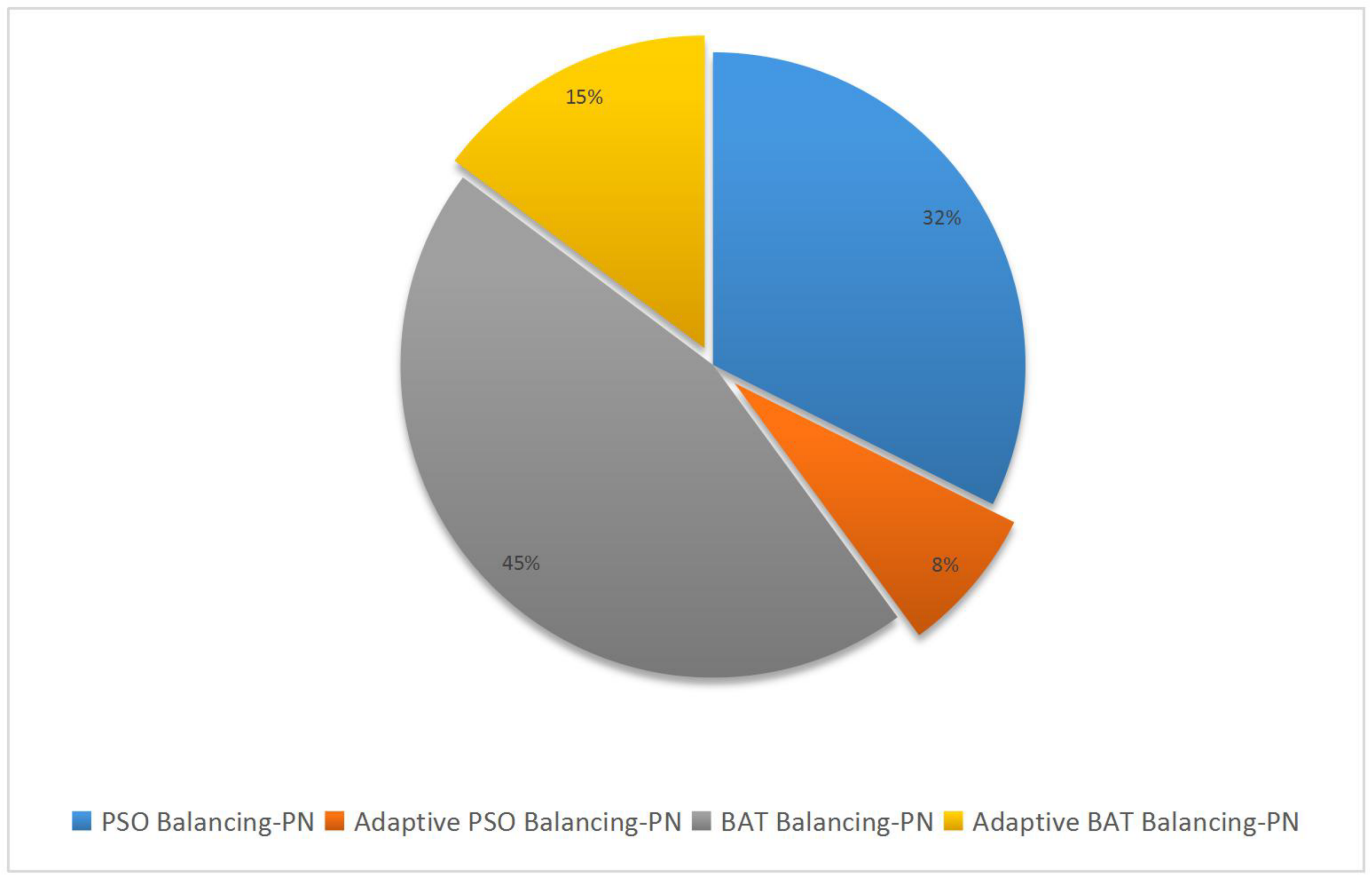
Average time of our four methods in experiment 2.

The Swarm Balancing Algorithms can save time, compared with the brute-force method using same dataset, when finding the best global parameters. For example, consider an imbalanced dataset with an imbalance ratio between majority class and minority class of 10, and the number of minority classes is 20. This means that *S* can be 9801 different values from 100 to 9900, and the scope of *K* is from 2 to 20. Therefore, the brute-force method will try a total of 186,219 combinations, requiring many repetitions to find the most suitable values of *S* and *K*. If we meet with a large and highly imbalanced dataset, the brute-force method will need to try many more possible combinations. It is clear that the Adaptive Swarm Balancing Algorithms can save more time. As Fig 5 shows, the Adaptive Swarm Balancing Algorithms only take one-third to one-fourth the time required by the Swarm Balancing Algorithms. Meanwhile, it is also easy to see that PSO is faster than the BA in the experiment. Therefore, with real-world data, the latter approach is better in performance, and it is more practical because it can flexibly process a large dataset in real time. Furthermore, application of the brute-force method to dataset 1608, the smallest of the six datasets, required 10279963.41876 *sec*. In comparison, the PSO Swarm Balancing, Adaptive PSO-Balancing, BA Swarm Balancing and Adaptive BA-Balancing algorithms required only 157.216446 *sec*., 93.580466, 172.0922 *sec*., and 123.9651 *sec*.

## Conclusion

Our methods clearly show their effectiveness in the processing of the imbalanced dataset classification problem with different dataset sizes. Meta-heuristic algorithms can blindly select the parameters of SMOTE to obtain a relatively high accuracy with a Kappa value that falls within the credible range. With changes in the sizes of the datasets, we used two methods to respectively improve processing of the normal-size imbalanced dataset and the large-size imbalanced dataset. The experiments indicate that the Swarm Balancing Algorithms are more suitable for a small dataset, and if we consider the big dataset as a data feed, the Adaptive Swarm Balancing Algorithms will more quickly and better solve the imbalance problem of the dataset. In the small- and normal-size datasets, no matter from which aspect is assessed, when compared with the neural network classification algorithm, PSO was better than BA. With large datasets however, except for search time, for which the PSO is still faster than BA, the other important performance parameters are better with BA rather than PSO. The Adaptive Swarm Balancing Algorithms operate more like a process of constant iteration and learning, which is more suitable to the actual problem in health and medical datasets. Because the number of diagnosed cases is constantly increasing daily, along with the gradual accumulation of cases, the dataset will grow into a large dataset that needs to be processed as a data feed. Therefore, the Adaptive Swarm Balancing Algorithms can effectively solve the imbalanced data classification problem in the large datasets typically found in the health and medical field. These methods will help the classifier to accurately classify and identify patient data.

## Supporting information

S1 Data Thoracic surgery in experiment 1. (CSV)

S2 Data Bioassay AID439 dataset in expeirment1. (CSV)

S3 Data Bioassay AID721 dataset in experiment 1. (CSV)

S4 Data Bioassay AID1284 dataset in experiment 1. (CSV)

S5 Data Bioassay AID362 dataset in experiment1 and 2. (CSV)

S6 Data Bioassay AID1608 dataset in experiment 2. (CSV)

S7 Data Bioassay AID746 dataset in experiment 2. (CSV)

S8 Data Bioassay AID687 dataset in experiment 2. (CSV)

S9 Data Bioassay AID456 dataset in experiment 2. (CSV)

S10 Data Bioassay AID373 dataset in experiment 2. (CSV)
